# Perspectives on the working hours of Australian junior doctors

**DOI:** 10.1186/1472-6920-14-S1-S13

**Published:** 2014-12-11

**Authors:** Nicholas J Glasgow, Michael Bonning, Rob Mitchell

**Affiliations:** 1 Medical School, Australian National University, Canberra ACT 0200, Australia; 2 Past Chair, Australian Medical Association, Council of Doctors-in-Training, Barton ACT 2604, Australia

## Abstract

The working hours of junior doctors have been a focus of discussion in Australia since the mid-1990s. Several national organizations, including the Australian Medical Association (AMA), have been prominent in advancing this agenda and have collected data (most of which is self-reported) on the working hours of junior doctors over the last 15 years. Overall, the available data indicate that working hours have fallen in a step-wise fashion, and AMA data suggest that the proportion of doctors at high risk of fatigue may be declining. It is likely that these changes reflect significant growth in the number of medical graduates, more detailed specifications regarding working hours in industrial agreements, and a greater focus on achieving a healthy work–life balance. It is notable that reductions in junior doctors’ working hours have occurred despite the absence of a national regulatory framework for working hours. Informed by a growing international literature on working hours and their relation to patient and practitioner safety, accreditation bodies such as the Australian Commission on Safety and Quality in Health Care (ACSQHC) and the Australian Medical Council (AMC) are adjusting their standards to encourage improved work and training practices.

## Introduction

As summarized elsewhere in this supplement, a significant body of evidence speaks to the negative impact of fatigue on both patient safety and the health and well-being of junior doctors. In Australia, the potential for harm was realized in the mid-1990s as a result of a growing body of international evidence. This resulted in a variety of campaigns and reforms that have sought to ameliorate the risks of unsafe working hours. Interventions have included workforce expansion, the development of more sophisticated rostering arrangements, greater emphasis on clinical handover procedures, and more detailed specifications regarding working hours in industrial agreements. Unlike countries in Europe and North America, Australia has not adopted a strict regulatory approach to working hours.

This article briefly outlines how postgraduate medical training operates in Australia, and then summarizes the available data describing working hour patterns in Australia for the past decade. The factors likely to shape junior doctor working hours over the next decade are also discussed.

## Postgraduate training in Australia

In Australia, postgraduate medical education comprises both pre-vocational and vocational stages.

The former begins with a one-year internship, which consists of several structured rotations through different specialties [1]. After successful completion of an accredited internship program, doctors are eligible for general registration with the Medical Board of Australia [2].

Pre-vocational training is usually completed by means of one to two years of generalist experience, predominantly in public hospitals. This stage of training is underpinned by the Australian Curriculum Framework for Junior Doctors, which outlines the knowledge, skills, and behaviours required to work safely in a variety of health care settings [3] and provides a structured bridge between undergraduate and specialty college curricula. At this stage of training, doctors are usually referred to as resident medical officers (RMOs).

Vocational training programs are administered by colleges, and typically require three to six years of supervised practice. Fellowship is awarded upon completion of training requirements. Like medical schools, college training programs operate within an accreditation framework managed by the Australian Medical Council (AMC). Doctors undertaking vocational training programs are usually referred to as “registrars.”

The hospital workforce also comprises those doctors who do not choose to specialize and who hold positions as career medical officers (CMOs). It is thought that up to 15% of graduates will not proceed to specialty training [4].

In this article, the term “junior doctor” refers to doctors in both pre-vocational and vocational training.

## Data on junior doctor working hours

Very few articles in the literature address the issue of working hours for Australian junior doctors. The few published papers point to the important links between working hours and safe practice and highlight the important role the medical profession has played in advancing this agenda in Australia [5-8]. Only a small number of national organizations have independently collected working hours data over the last decade, and most published data have relied on self-reporting, which has inherent methodological limitations.

### Australian Medical Association

The Australian Medical Association (AMA) launched its Safe Working Hours campaign in 1995. This included the commissioning and reviewing of research in topic areas that included the actual working hours of junior doctors, systems of work, and the effect of fatigue on learning and performance. These efforts culminated in the development of a national code of practice around working hours, shift work, and rostering for hospital doctors [9]. This process was supported by the Australian government.

Advocacy by the AMA has emphasized the need to avoid absolute, enforceable limits on single elements such as the maximum length of a safe shift or the break required between episodes of work. This is because there are multiple determinants of fatigue, including quantity of night work, shift length, on-call commitments, and access to breaks [9,10].

The AMA has audited the work patterns of hospital doctors every five years since 2001 [11-13]. Each self-reporting survey was conducted during a designated audit week and utilized a risk-assessment calculator based on the national code of practice and validated in Australasian settings [10,14]. The model allocates doctors to one of three risk categories on the basis of various determinants of fatigue. The latest audit, completed in August 2011, includes data from 1,399 hospital-based doctors.

**Figure 1:**
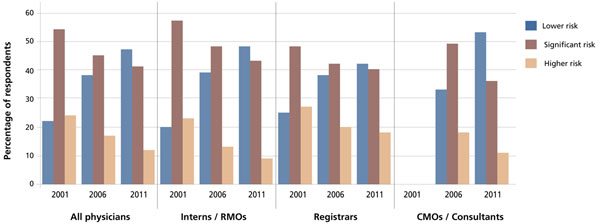
Distribution of fatigue-related risk categories among hospital doctors in Australia, by training classification

**Figure 2:**
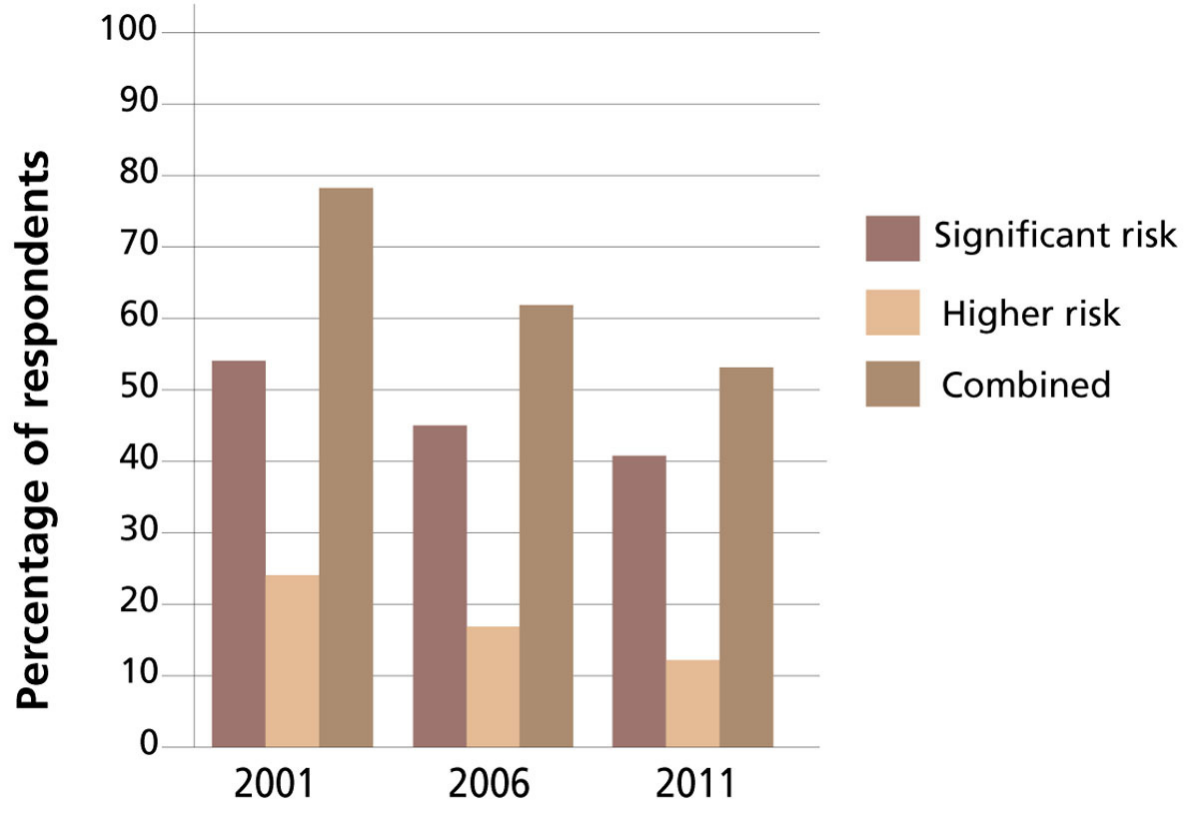
Distribution of higher fatigue-related risk categories among hospital doctors in Australia, for all training classifications combine

The distribution of the three risk categories across the three AMA audits, stratified by training classification, is shown in Figure 1. Figure 2 shows the distribution of the higher-risk categories across the three AMA audits for all training classifications combined.

The audit results show a consistent, step-wise decline in the proportion of doctors in the higher-risk categories. Note should be made of the fact that the total number of respondents in the higher-risk category was halved between 2001 and 2011. The average weekly working hours of surveyed doctors in 2001 was 61.3; it fell to 55.1 in 2011.

The complete data for the three studies also show improvements across “at-risk” disciplines, such as Surgery and Obstetrics and Gynaecology. The latter has improved markedly, with 74% of respondents falling into the lower-risk category (as compared to 28% in 2006 and 7% in 2001) [13]. Internal Medicine has also seen improvements in its risk profile, with 46% in the lower-risk category (as compared with 36% in 2006 and 14% in 2001) [13]. The improvements for the surgical category are also encouraging given the high baseline reported in 2001. This may be the result of efforts by the surgical community to publicly recognize the impact of fatigue and to address cultural and systemic issues [15].

While there has been some steady and consistent improvement in working practices, the 2011 audit demonstrates that this has not been universal. For instance, one in five doctors reported that they worked every day of the seven-day audit period. For one individual, this included a shift of 43 hours. It is also of concern that one respondent declared that he or she worked 120 hours in the audit week [13].

### Australian Institute of Health and Welfare

The Australian Institute of Health and Welfare (AIHW) collects data on working practices and employment trends for the medical workforce at the time of medical registration renewal. These data are published annually as Medical Labour Force Surveys.

The latest AIHW data on total working hours confirm the trend over the past decade of reduced working hours for doctors [16,17]. Comparison of annual data from this period reveals a decrease in the average weekly working hours for non-GP specialists, from 48 in 1999 to 45.7 in 2005 and 44.4 in 2010. The corresponding figures for registrars were 51.7, 49.1, and 49.9 hours. No specific data are available for RMOs and interns.

Some of this reduction has been attributed to the increasing number of women in the physician workforce, who tend to work fewer hours than their male counterparts [17]. The average weekly working hours of all female physicians in the period 1999–2009 remained stable at approximately 38 hours. For men, the average number of weekly working hours decreased by approximately 4 hours per week to 44.9.

### Medicine in Australia: Balancing Employment and Lifestyle (MABEL) [18]

Further corroborating evidence of the reduction in working hours is found in MABEL, an ongoing national longitudinal survey of doctors funded by the National Health and Medical Research Council [19]. The 2008 survey received 924 complete responses from interns and RMOs. Analysis showed that pre-vocational trainees worked, on average, 50.7 hours per week. The 1,072 responses from registrars showed that this group worked, on average, 47.1 hours per week [20]. The survey results also showed that male doctors tended to work more hours than their female counterparts; this is consistent with data from other sources [17].

## Factors likely to influence working hours over the next decade

### Increasing numbers of medical graduates

Over the last decade both the number of medical schools and the number of commencing medical students has increased significantly in response to an undersupply of medical graduates that dates to 1980. Since 2002, the number of medical schools has increased from 10 to 18. As well, the total number of commencing medical students has more than doubled, with the intake increasing by 2,110 (or 127.1%) between 2000 and 2011, for a total of 3,770 students. By 2016 it is projected that the number of graduates will increase to 3,970 – an increase of 65.9% from 2009 and 183% from 1999 [21].

State and territory governments have responded by increasing the number of postgraduate training places and are actively changing the traditional patterns of employment, in part by reducing the number of overtime shifts. As evidence of this, the number of advanced training positions for specialty trainees has increased by nearly 50% in the last five years [21]. Although this may be expected to reduce hours of work in the medium term, a recent report to Health Workforce Australia (a Commonwealth agency) that contrasted jurisdictions with increased numbers of graduates with jurisdictions with stable numbers of graduates suggests that, at this stage, "there are few consistent changes in the working patterns of junior doctors and their supervisors associated with graduate expansion [22].”

### Emphasis on work–life flexibility

Although significant anecdotal evidence suggests that flexible workplace arrangements are becoming more prevalent, specific data describing this trend are limited. MABEL [21] and the AMA [23] have both conducted self-reporting surveys to determine the drivers of demand for flexible workplace arrangements. Two of the key factors identified were demographic changes and a cultural trend within medicine to recognize the value of time away from the workplace. AMA data from 2007 showed that 85% of doctors surveyed were anticipating a need for flexible arrangements during their next 10 years of practice, and this figure is predicted to increase.

### Health reform

The Australian government is prosecuting a major health reform agenda, central to which are mechanisms to achieve a greater focus on transparency, quality, safety, and the minimization of risk. The Australian Commission for Safety and Quality in Health Care (ACSQHC) has recently produced its 2011 report to the Australian Government drawing “attention to the importance, and challenges, of implementing improvements to the safety and quality of health care.” Chapter four – "Developing a positive safety culture” – addresses the safety culture, (including stress recognition) of health care organizations. It explicitly considers working hours and the potential negative impact of prolonged working hours on the safety and quality of health care delivery [24].

### Medical curricula and training standards

Despite the fact that the curricula are different in the 18 universities that offer primary medical education qualifications in Australia, all schools must be accredited against AMC standards. In an explanatory note for Standard 7.3, the AMC states “Attention should be paid to the time commitment required of medical students, especially during the clinical years. Students’ expected hours for course participation should be determined taking into account the effects of fatigue and sleep deprivation on learning, clinical activities, and student health and safety [25].”

The AMC is currently reviewing its standards for implementation in 2013. From the consultative process, it is clear that the new standards will have an enhanced focus on quality and safety in health care. It is expected that this will result in a greater focus on working hours in undergraduate curricula.

Like medical schools, college training programs are also accredited against AMC standards. Standard 1.4 – “Interaction with the health sector” – has an explanatory note that states “The duties, working hours and supervision of trainees should be consistent with the delivery of high quality, safe patient care. Ensuring trainees can meet their educational goals and service delivery requirements within safe hours of work is the responsibility of all parties [26].”

## Discussion

The working hours of junior doctors have been the subject of discussion in Australia since the mid-1990s. As described above, surveys of working hours suggest a small but significant reduction in overall working hours from the early 2000s. In some disciplines, particularly Surgery, hours of work have shown relative improvement from a high baseline. However, the available data have significant limitations, which derive largely from their reliance on self-reporting.

The complexity of improving safety in health care with regards to minimizing fatigue has been described in international literature for many years [27,28]. In Australia, profession-led advocacy promoting risk minimization as well as patient and staff safety has been key to lowering the risk of fatigue without imposing maximum working hours regulations [9]. Early acknowledgement of the implications for quality of care has also enabled reform [8]. Appropriate medical staffing, along with proper patient handover and fatigue management strategies, has been recognized as essential to ensuring that continuity of patient care is not compromised.

Training and service delivery are inextricably linked. Although a 2010 systematic review failed to identify an overall negative impact from capped working hours during training, trainees in various settings have raised concerns that reduced working hours will have an impact on the quality of their clinical education [29,30]. In Australia, surgical registrars perceive that practising approximately 60 hours per week provides an appropriate volume of working hours for surgical training; however, study and lifestyle demands are better accommodated by working approximately 55 hours per week [31]. Reports from other settings have suggested that it will be necessary to explore alternate educational strategies in order to mitigate the impact of reduced working hours on training opportunities [32]. On this basis, Australian commentators have suggested that more sophisticated rostering arrangements are required to preserve quality in clinical education [13].

In the context of a more than doubling in the number of medical graduates, there is pressure on Australian governments to resource adequate postgraduate training positions. In addition to these workforce changes, the ACSQHC’s explicit emphasis on developing a safe workplace culture and the development of new safety and quality accreditation standards provide regulatory pressure. It is anticipated that enhanced national emphasis on safety and quality will be reflected in the revised AMC accreditation standards, which, in turn, will be more explicitly referenced in training curricula.

The Australian experience suggests that working hour reform is assisted by the effective engagement of government agencies, health services, training providers, and the medical profession. It is possible that the parallel efforts of these groups have obviated the need for national working hour regulations. These reflections may be of relevance to other jurisdictions as they consider approaches to minimizing trainee and physician fatigue.

## Conclusion

Available data suggests that the working hours of Australian junior doctors may be decreasing, with a parallel reduction in the proportion of practitioners at significant risk of fatigue. These improvements, which can be attributed to a number of factors, have been possible in the absence of a strict regulatory framework for working hours. The impact on patient safety and postgraduate training has yet to be thoroughly evaluated.

Looking forward, workforce expansion, increasing emphasis on work–life balance, curricular changes, and national safety and quality reforms will continue to drive change. To achieve the best possible outcomes for patients and physicians, ongoing reform of work practices will need to be supported by additional resources for the workforce, training, and handover.

## Authors' contributions

NJG proposed the content and wrote the first drafts of the paper after discussion with the other authors. MAB and RM collected the working hours data and edited the drafts.

## Competing interests

The authors declare that they have no competing interests.

## Declarations

Resources and secretariat support for this project was provided by the Royal College.

This article has been published as part of *BMC Medical Education* Volume 14 Supplement 1, 2014: Resident duty hours across borders: an international perspective. The full contents of the supplement are available online at http://www.biomedcentral.com/bmcmededuc/supplements/14/S1. This article was submitted in 2011 and peer reviewed in 2012. Final acceptance for publication as part of this supplement was in 2014.

Publication of this supplement was supported and funded by the Royal College of Physicians and Surgeons of Canada. The funding agency played no role in the design, in the collection, analysis and interpretation of data; in the writing of the manuscripts; and in the decision to submit the manuscript for publication. The articles have been through the journal's standard peer review process for supplements. The Supplement Editors declare that they have no competing interests.
